# Pgam5 aggravates hyperglycemia-induced myocardial dysfunction through disrupting Phb2-dependent mitochondrial dynamics

**DOI:** 10.7150/ijms.92872

**Published:** 2024-05-05

**Authors:** Yingzhen Chen, Jungang Huang, Hao Zhou, Jianguo Lin, Jun Tao

**Affiliations:** 1 Department of Anesthesiology, Sun Yat-sen Memorial Hospital, Sun Yat-sen University, Guangzhou, China.; 2 Department of Cardiovascular Surgery, Sun Yat-sen Memorial Hospital, Sun Yat-sen University, Guangzhou 510120, China.; 3 Guang'anmen Hospital of Chinese Academy of Traditional Chinese Medicine, Beijing, China.

**Keywords:** Pgam5, Phb2, mitochondrial fission, mitophagy, diabetic cardiomyopathy

## Abstract

This study aims to elucidate the roles of Phosphoglycerate Mutase Family Member 5 (Pgam5) and Prohibitin 2 (Phb2) in the context of hyperglycemia-induced myocardial dysfunction, a critical aspect of diabetic cardiomyopathy. The research employed primary cardiomyocytes, which were then subjected to hyperglycemia treatment to mimic diabetic conditions. We used siRNA transfection to knock down Pgam5 and overexpressed Phb2 using adenovirus transfection to assess their individual and combined effects on cardiomyocyte health. Mitochondrial function was evaluated through measurements of mitochondrial membrane potential using the JC-1 probe, and levels of mitochondrial reactive oxygen species (ROS) were assessed. Additionally, the study involved qPCR analysis to quantify the transcriptional changes in genes related to mitochondrial fission and mitophagy. Our findings indicate that hyperglycemia significantly reduces cardiomyocyte viability and impairs mitochondrial function, as evidenced by decreased mitochondrial membrane potential and increased ROS levels. Pgam5 knockdown was observed to mitigate these adverse effects, preserving mitochondrial function and cardiomyocyte viability. On the molecular level, Pgam5 was found to regulate genes associated with mitochondrial fission (such as Drp1, Mff, and Fis1) and mitophagy (including Parkin, Bnip3, and Fundc1). Furthermore, overexpression of Phb2 countered the hyperglycemia-induced mitochondrial dysfunction and normalized the levels of key mitochondrial antioxidant enzymes. The combined data suggest a protective role for both Pgam5 knockdown and Phb2 overexpression against hyperglycemia-induced cellular and mitochondrial damage. The study elucidates the critical roles of Pgam5 and Phb2 in regulating mitochondrial dynamics in the setting of hyperglycemia-induced myocardial dysfunction. By modulating mitochondrial fission and mitophagy, Pgam5 and Phb2 emerge as key players in preserving mitochondrial integrity and cardiomyocyte health under diabetic conditions. These findings contribute significantly to our understanding of the molecular mechanisms underlying diabetic cardiomyopathy and suggest potential therapeutic targets for mitigating myocardial dysfunction in diabetes.

## Introduction

Diabetic cardiomyopathy, a significant complication of diabetes, presents with distinct epidemiological, diagnostic, and pathophysiological features 1, 2. Epidemiologically, it is increasingly prevalent in parallel with the global rise in diabetes. Diagnostically, it is identified through a combination of cardiac imaging, biomarkers, and functional assessments, often in the absence of conventional cardiovascular risk factors 3, 4. Pathophysiologically, diabetic cardiomyopathy is characterized by metabolic disturbances, myocardial fibrosis, and impaired calcium handling, influenced by hyperglycemia, insulin resistance, and lipotoxicity 5, 6. This complex interplay contributes to the altered myocardial structure and function observed in patients.

In diabetic cardiomyopathy, mitochondrial dysfunction is a central pathophysiological mechanism. This condition involves altered mitochondrial dynamics, impaired bioenergetics, and increased production of reactive oxygen species (ROS) 7, 8. Hyperglycemia and insulin resistance, hallmarks of diabetes, contribute to mitochondrial dysfunction, which in turn affects cardiac cell metabolism and survival 9, 10. The disruption of mitochondrial homeostasis leads to cardiomyocyte apoptosis, fibrosis, and ultimately, cardiac dysfunction 11, 12. This complex interplay underscores the need for targeted therapeutic strategies addressing mitochondrial health in the management of diabetic cardiomyopathy.

Phosphoglycerate Mutase Family Member 5 (Pgam5) plays a pivotal role in metabolic regulation, especially within the mitochondria 13. Functionally distinct from traditional phosphoglycerate mutases, Pgam5 is involved in modulating mitochondrial dynamics, which includes the processes of mitochondrial fission and fusion 14, 15. These dynamics are crucial for maintaining cellular energy homeostasis. Moreover, Pgam5 is implicated in the regulation of mitochondrial-induced cell death pathways, linking it to various metabolic and neurodegenerative diseases 16, 17. Its involvement in these pathways highlights its potential as a therapeutic target in disorders characterized by mitochondrial dysfunction.

In the realm of mitochondrial biology, Prohibitin 2 (Phb2) emerges as a pivotal protein with profound implications in the pathophysiology of diabetes. Its cardinal function lies in preserving the integrity and functionality of mitochondria, components vital for metabolic processes and insulin sensitivity 18, 19. In the context of diabetes, a disruption in Phb2's regulation could instigate mitochondrial malfunctions, precipitating insulin resistance and metabolic anomalies 20, 21. Given Phb2's integral part in orchestrating mitochondrial dynamics and its nexus with essential metabolic pathways, it presents an intriguing prospect as a therapeutic conduit for managing diabetes. This approach would emphasize the rejuvenation of mitochondrial health and reestablishment of metabolic equilibrium. Our research endeavors to elucidate the roles of Pgam5 and Phb2 in modulating the evolution of diabetic cardiomyopathy and its underlying mechanisms.

## Methods

### Cultivation and intervention of cells

The procedures for cultivating and intervening cells were executed in the following manner. Adult Rat Ventricle Myocytes (ARVMs) were propagated in Dulbecco's Modified Eagle Medium enriched with high glucose (DMEM-HG) (Gibco, USA). This medium was further fortified with 10% (v/v) fetal bovine serum (FBS) (Gibco, USA) and a 1× (50 unit/ml) concoction of penicillin-streptomycin (P/S) (Gibco, USA). To simulate hyperglycemic damage, the cardiomyocytes were subjected to a high-glucose DMEM environment (containing 30 mmol/l of glucose) in conjunction with 20% fetal bovine serum, maintained for an interval of 48 hours.

### Cardiomyocyte contractility measurements

In the realm of cardiomyocyte contractility investigations, an IonOptix Fluorescence and Contractility System (IonOptix, MA, USA) was employed for the acquisition of data in cardiomyocytes triggered at a field-stimulation rate of 1 Hz, replicating a methodology previously documented. To instigate contractions, rectangular depolarizing pulses of 2 ms duration and with an intensity twice that of the diastolic threshold, were disseminated via platinum electrodes. The process of cell shortening was monitored by utilizing edge track detection, whilst calcium transients were quantified via epifluorescence following the infusion of cardiomyocytes with 1 μmol/l of Fura-2 AM (#F1225, Invitrogen) for a duration of 10 minutes. The IonWizard software (IonOptix, MA, USA) was used to evaluate the data, specifically targeting 5 to 10 sequential single-cell contractions during a steady-state period for detailed analysis.

### ROS detection

The quantification of intracellular reactive oxygen species (ROS) was executed employing the Beyotime ROS Assay Kit (S0033S, China), as per the guidelines provided by the manufacturer. Specific treatments were applied to the HL-1 cells, which were subsequently incubated in a fresh medium with the addition of 10 μM DCFHDA at a constant temperature of 37 °C for a duration of 30 minutes. Post incubation, the cells underwent comprehensive cleansing before being gathered for subsequent examination.

### siRNA

In the present investigation, we utilized a Pgam5-specific siRNA set, composed of three distinct siRNAs, each with a length of 20-25 nucleotides, procured from Santa Cruz Biotechnology (Santa Cruz, CA). We adhered to well-established procedures for cellular transfection with the Pgam5 siRNA. A non-targeting scramble siRNA served as the control. Assurance of successful Pgam5 siRNA transfection was acquired via Western blot analysis, as detailed in 22.

### Adenovirus

The adenovirus Phb2 (Catalog No. sc-419296-LAC) was obtained from Santa Cruz Biotechnology, Inc. and subjected to conventional methods for purification and infectious virus titer determination, as per reference 23. The process commenced with the infection of AD-293T cells using 2 pfu/cell in serum-free DMEM at 37°C for an hour, facilitating adenovirus production. Subsequently, the infective medium was substituted, and the cells were nurtured in DMEM fortified with 5% horse serum until the onset of comprehensive cell lysis, generally within a span of 72 hours. To enhance virion liberation, the virion-laden medium underwent a triad of freeze-thaw cycles ensuring total cell lysis. The aftermath was a supernatant that was subjected to centrifugation at 3000 rpm for a half-hour duration at 4°C, thereby eliminating cellular detritus. The supernatant was then partitioned into aliquots and preserved at -80°C. For infecting ARVM, two successive infection cycles were executed [5 hours and overnight (O/N)] utilizing an MOI of 300 in 1 ml of fully supplemented culture medium, in line with reference 24.

### Mitochondrial membrane potential (MMP) detection

The process of assessing MMP was performed by subjecting cells to a single concentration of JC-1 working solution (Meilunbio) at a controlled temperature of 37°C for a duration of 20 minutes. Post incubation, the cells underwent a dual rinse using a chilled 1× buffer solution (Meilunbio) and were immediately visualized under a Leica microscope. The corresponding MMP was determined via ImageJ, employing the computation of the mean fluorescence intensity ratio derived from red and green fluorescence.

### Western blot analysis

In the exploration of protein analysis, Western blotting was employed. The process commenced with the extraction of cellular protein, which was achieved by employing RIPA lysis buffer fortified with a protease inhibitor mixture and PhosSTOP, both products of Roche. Following total homogenization under chilled conditions and subsequent centrifugation, the protein extract was amalgamated with a loading buffer and segregated on 4%-12% NuPAGE Bis-Tris gels, as per the methodology detailed in 25. The partitioned proteins were subsequently transferred to a nitrocellulose membrane. A blocking step was then carried out using a 5% nonfat milk solution in a Tris-buffered saline environment. The membranes were then subjected to an overnight incubation at 4°C with primary antibodies. Following this, the membranes were subjected to a series of four washes, each lasting five minutes, using TBST buffer. This was succeeded by an incubation period with a horseradish peroxidase-linked secondary antibody, carried out at ambient temperature for a duration of one hour. Following another washing sequence, the membrane was developed using an enhanced chemiluminescence reagent from Invitrogen, and the Fluor Chem M System from ProteinSimple, San Jose, CA, USA, was employed for signal detection. Protein expression was quantified and standardized against either GAPDH or β-Actin, serving as internal controls. The signal density was further scrutinized using ImageJ software.

### RNA Isolation and Real-Time PCR

Cellular RNA underwent a purification process by employing Trizol reagent (Invitrogen), in adherence to the guidelines provided by the manufacturer. Following this, the conversion of RNA into cDNA was facilitated by the SuperScript IV reverse transcriptase, utilizing random hexamers. Quantitative PCR, employing SYBR green (Bio-Rad), was subsequently carried out, with the specifics of the primer outlined in Supplementary Table S1. The extent of mRNA in each specimen was standardized to either Gapdh or β-actin RNA levels.

### ELISA

In the conducted experiments, the enzymatic functions of catalase, glutaredoxin, thioredoxin reductase (TrxR), and peroxiredoxin were evaluated. The evaluation process employed commercially available assay kits procured from BioSino Bio-Technology & Science Inc., based in China. The experimental procedures were conducted in strict adherence to the guidelines provided by the manufacturer 26.

### Statistical analysis

Values are articulated as the average ± standard uncertainty (SEM), with statistical relevance established at P<0.05. To evaluate statistical importance, we utilized either the unpaired Student's t-test or a one-way variance analysis (ANOVA). When the gene of interest exhibited no initial expression, we opted for relative expression as a substitute for fold change. Reference groups were classified as either non-treated, treated with a vehicle, or subjected to sham operations. All statistical evaluations were carried out via the GraphPad Prism software.

## Results

### Pgam5 knockdown attenuates the hyperglycemia-mediated cardiomyocyte death and dysfunction

**Figure 1:**
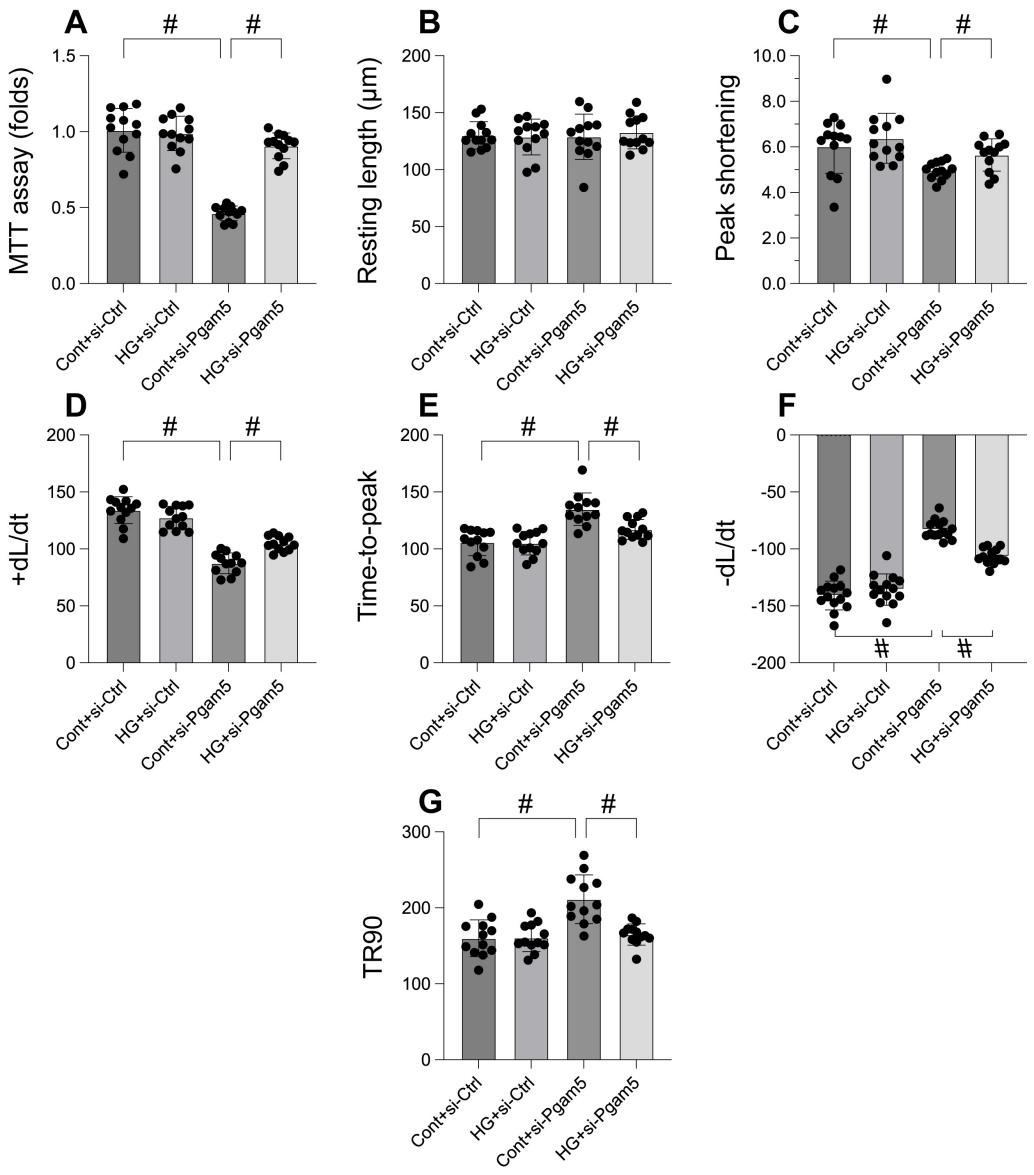
** Pgam5 knockdown attenuates the hyperglycemia-mediated cardiomyocyte death and dysfunction. A.** Cell viability was determined by MTT assay. **B-G.** Pgam5 siRNA was transfected into cardiomyocytes and then the contractile was measured, including resting length remained, peak shortening (PK), maximal velocity of shortening (dL/dt), and time-to-peak shortening, maximal velocity of relengthening (-dL/dt), and time-to-90% relengthening (TR90). #p<0.05.

In an effort to elucidate the role of Pgam5 in hyperglycemia-induced cardiomyocyte impairment and mortality, we embarked on a rigorous investigation employing primary cardiomyocytes. Pgam5 siRNA transfection was performed on these cells before they were subjected to a 48-hour hyperglycemic condition. Employing the MTT assay to gauge cell viability (Figure 1A), we discovered that hyperglycemia notably reduced viability, a repercussion that was tempered by Pgam5 siRNA. We then proceeded to measure contractile parameters in isolated cardiomyocytes. Despite the consistency in resting length across all treatments, hyperglycemia compromised contractility, as underscored by the decline in peak shortening, maximal shortening velocity, and time to reach peak shortening (Figure 1B-G). Altered relaxation kinetics were also observed, characterized by diminished maximal velocity of relengthening and elongated time to achieve 90% relengthening (Figure 1B-G). Remarkably, transfection with Pgam5 siRNA alleviated both these contractile and relaxation deficits (Figure 1B-G). Taken together, our findings strongly indicate a protective function of Pgam5 deletion in cardiomyocytes against hyperglycemic damage.

### Mitochondrial dysfunction was induced by hyperglycemia due to increased Pgam5

**Figure 2:**
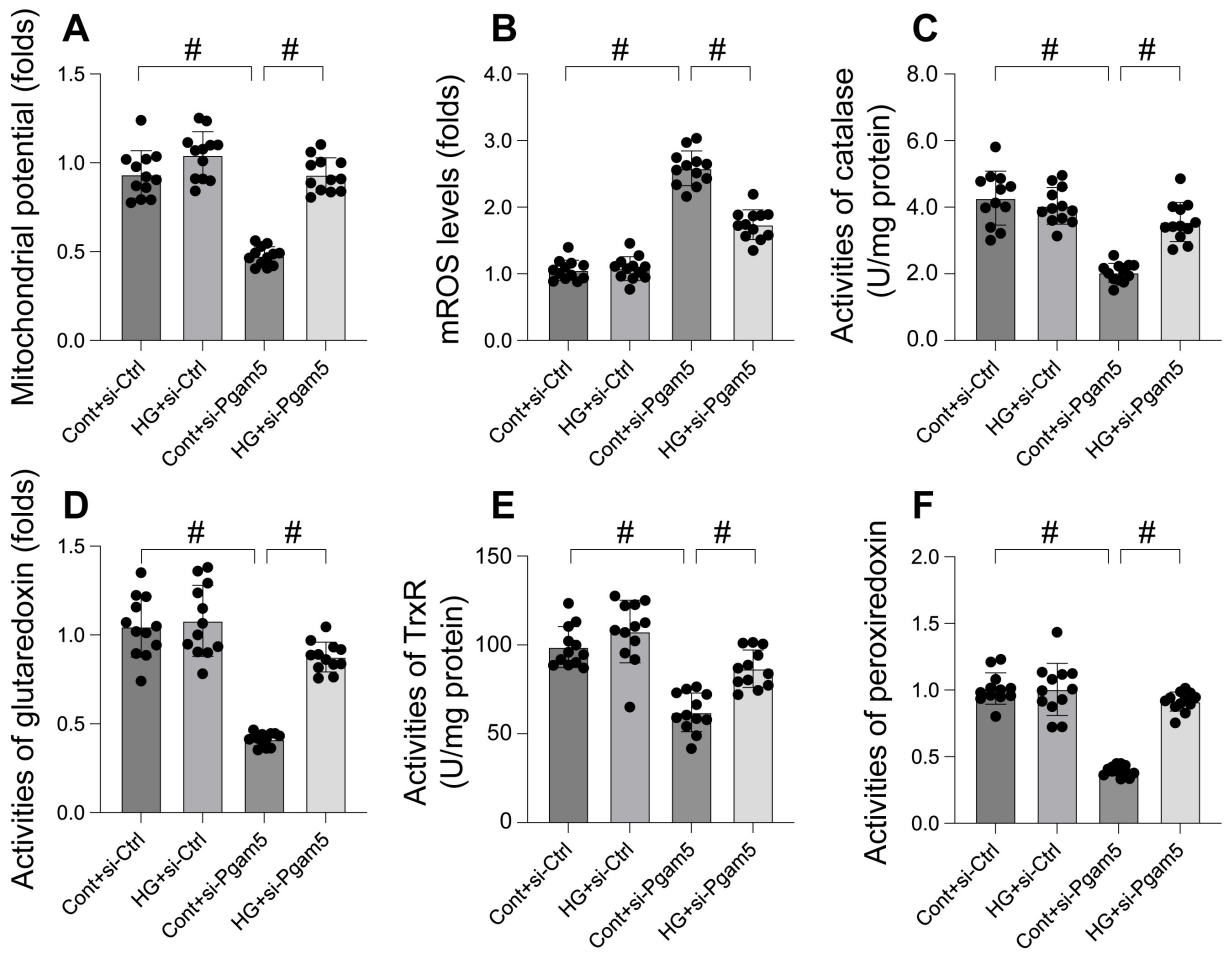
** Mitochondrial dysfunction was induced by hyperglycemia due to increased Pgam5. A.** JC-1 probe was used to stain mitochondrial membrane potential. B. ROS production was measured in cardiomyocytes. **C-F.** The activities of catalase, glutaredoxin, thioredoxin reductase (TrxR), and peroxiredoxin were determined by ELISA. #p<0.05.

Mitochondrial dysfunction is increasingly recognized as a key mechanism in hyperglycemia-induced cardiomyocyte damage. This study aimed to elucidate the roles of Pgam5 and Phb2 in regulating mitochondrial impairment under hyperglycemic conditions. Mitochondrial membrane potential, a critical indicator of mitochondrial function, was assessed using the JC-1 probe. Our results, depicted in Figure 2A, indicate that hyperglycemia significantly suppressed mitochondrial membrane potential in cardiomyocytes, an effect mitigated by Pgam5 siRNA transfection. Furthermore, we observed a marked increase in mitochondrial reactive oxygen species (mROS) levels following hyperglycemia treatment (Figure 2B), which was notably absent in cells transfected with Pgam5 siRNA. Additionally, the study examined the levels of key mitochondrial antioxidant enzymes, including catalase, glutaredoxin, thioredoxin reductase (TrxR), and peroxiredoxin (Figure 2C-F). These enzymes, crucial for combating oxidative stress, were found to be downregulated in the hyperglycemia-exposed cardiomyocytes. Intriguingly, Pgam5 siRNA transfection restored these antioxidant enzyme levels to near-normal (Figure 2C-F). Collectively, our findings indicate that Pgam5 plays a significant role in the hyperglycemia-induced mitochondrial dysfunction pathway, suggesting a novel therapeutic target for mitigating cardiomyocyte damage in hyperglycemic conditions.

### Mitophagy was activated whereas mitochondrial fission was inhibited by Pgam5 deletion in the setting of hyperglycemia

**Figure 3:**
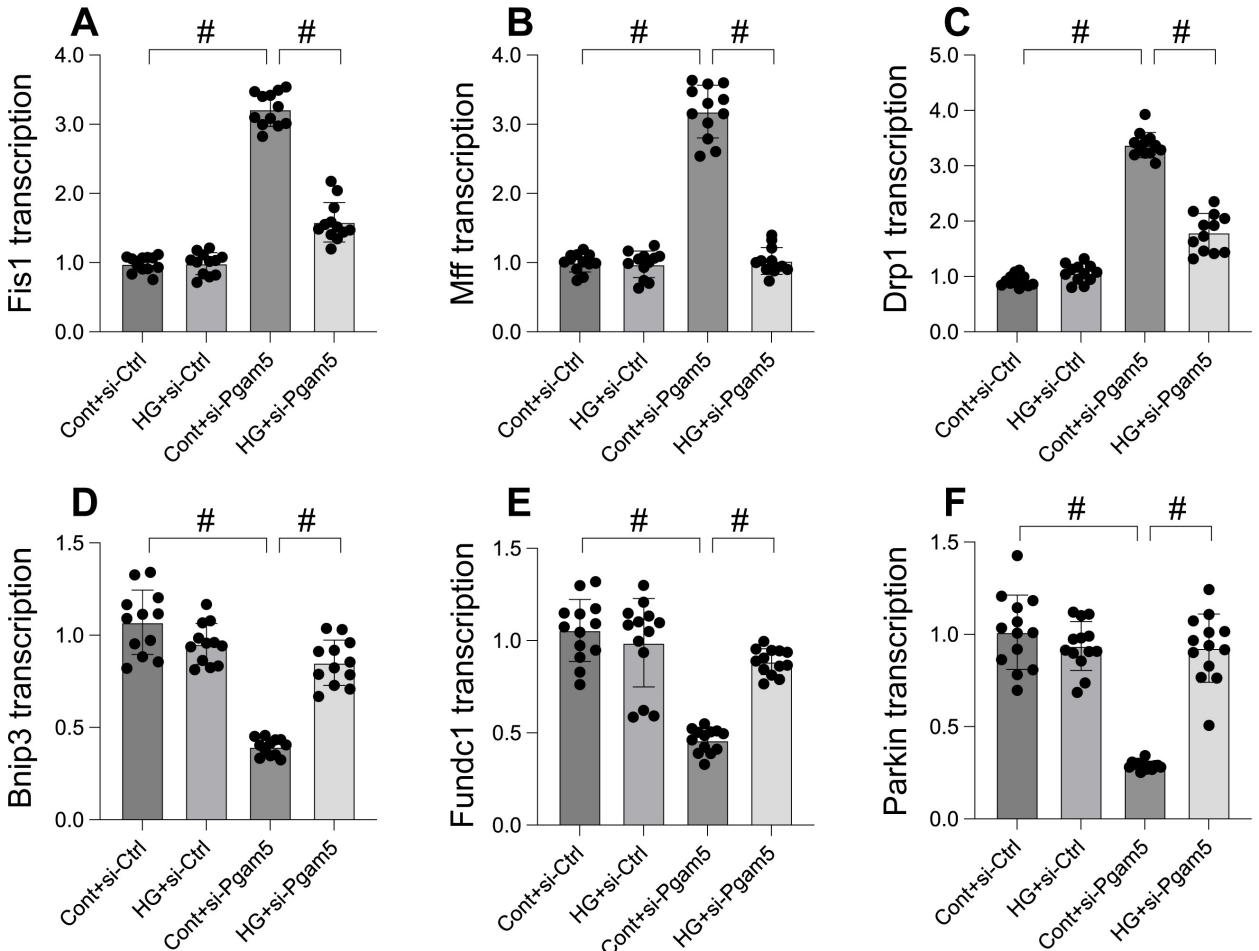
** Mitophagy was activated whereas mitochondrial fission was inhibited by Pgam5 deletion in the setting of hyperglycemia. A-C.** RNA was isolated from cardiomyocytes and then the transcription of Drp1, Mff, and Fis1 was measured via qPCR. **D-F.** RNA was isolated from cardiomyocytes and then the transcription of Parkin, Fundc1, and Bnip3 was measured via qPCR. #p<0.05.

Mitochondrial dysfunction, a critical factor in cellular health, is intricately linked to processes like mitochondrial fission and mitophagy. This study explores the impact of Pgam5 deletion on these processes under hyperglycemic conditions. Through qPCR analysis, we observed an upregulation in genes associated with mitochondrial fission (Drp1, Mff, and Fis1) following hyperglycemia treatment (Figure 3A-C). Concurrently, genes essential for mitophagy, including Parkin, Bnip3, and Fundc1, were significantly downregulated (Figure 3D-F). Notably, Pgam5 deletion reversed these trends, reducing the expression of fission-related genes (Figure 3A-C) while enhancing the transcription of mitophagy-related genes (Figure 3D-F). These results suggest a dual role of Pgam5 in modulating mitochondrial fission and mitophagy, particularly in the context of hyperglycemic stress in cardiomyocytes.

### Pgam5 controlled mitophagy and mitochondrial fission through Phb2

**Figure 4:**
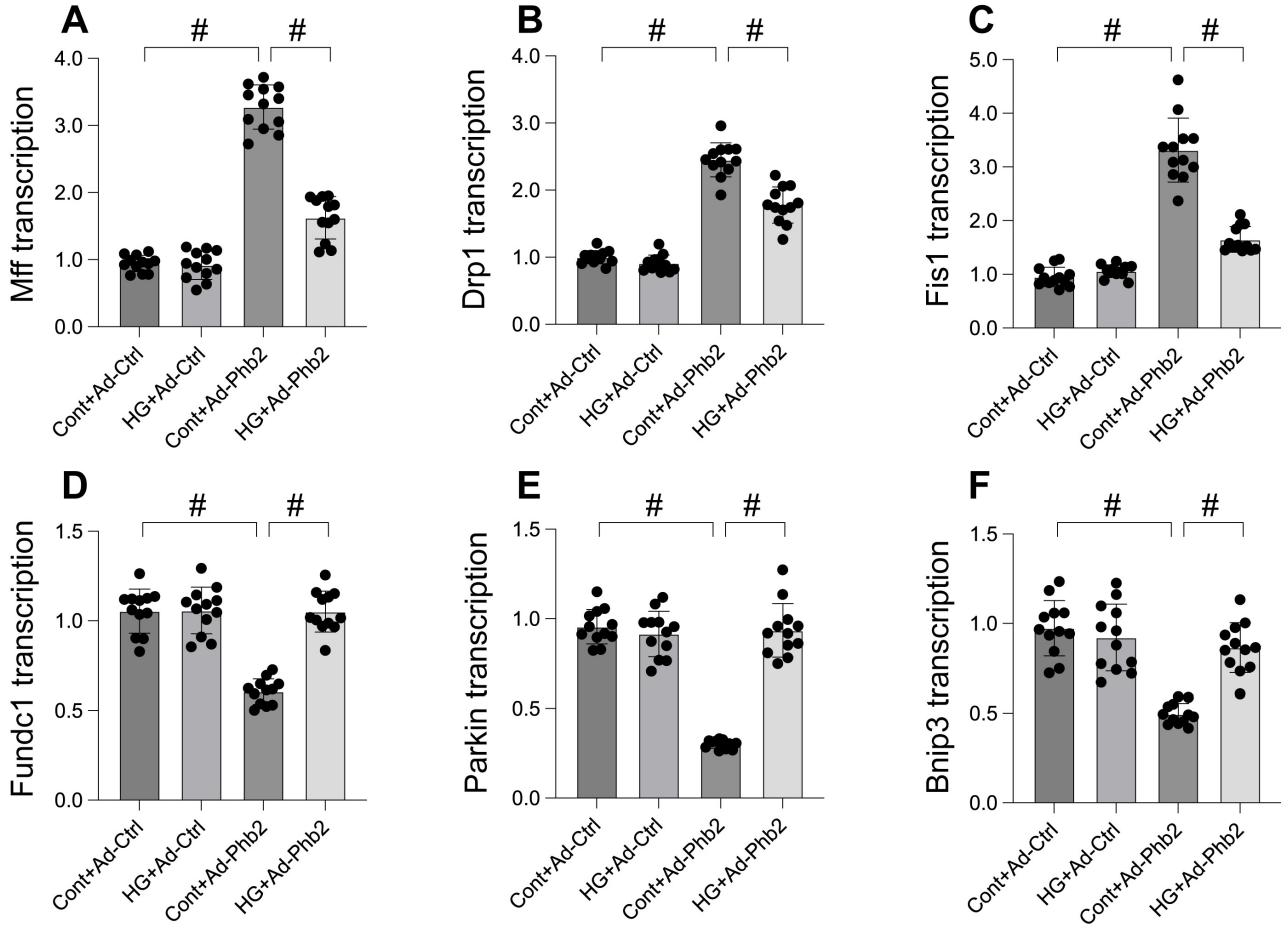
** Pgam5 controlled mitophagy and mitochondrial fission through Phb2. A-C.** RNA was isolated from cardiomyocytes and then the transcription of Drp1, Mff, and Fis1 was measured via qPCR. Phb2 adenovirus were transfected into cardiomyocytes before hyperglycemia treatment. **D-F.** RNA was isolated from cardiomyocytes and then the transcription of Parkin, Fundc1, and Bnip3 was measured via qPCR. Phb2 adenovirus were transfected into cardiomyocytes before hyperglycemia treatment. #p<0.05.

Our study delved into the molecular mechanisms of Pgam5's influence on mitochondrial fission and mitophagy, focusing on Phb2, a protein crucial for maintaining mitochondrial membrane integrity. Initially, we observed a significant downregulation in Phb2 transcription upon hyperglycemia exposure, which was countered by Pgam5 deletion, thereby sustaining Phb2 abundance. To probe the association between Phb2 and Pgam5-mediated mitochondrial dynamics, we transfected siRNA against Phb2 into Pgam5-silenced cardiomyocytes prior to hyperglycemia treatment. qPCR analysis revealed that Pgam5 siRNA transfection led to the downregulation of hyperglycemia-induced Drp1, Mff, and Fis1 expression (Figure 4A-C). However, the introduction of Phb2 siRNA nullified the inhibitory effects of Pgam5 deletion on mitochondrial fission (Figure 4A-C). Similarly, while Pgam5 deletion maintained the transcription of Parkin, Fundc1, and Bnip3 (Figure 4D-F), this effect was abrogated by Phb2 siRNA in hyperglycemia-treated cardiomyocytes. Overall, our data suggest a regulatory pathway where Pgam5 modulates mitochondrial fission and mitophagy via Phb2, offering insights into potential therapeutic targets for hyperglycemia-induced cardiomyocyte dysfunction.

### Phb2 overexpression reduced mitochondrial dysfunction upon hyperglycemia stress

**Figure 5:**
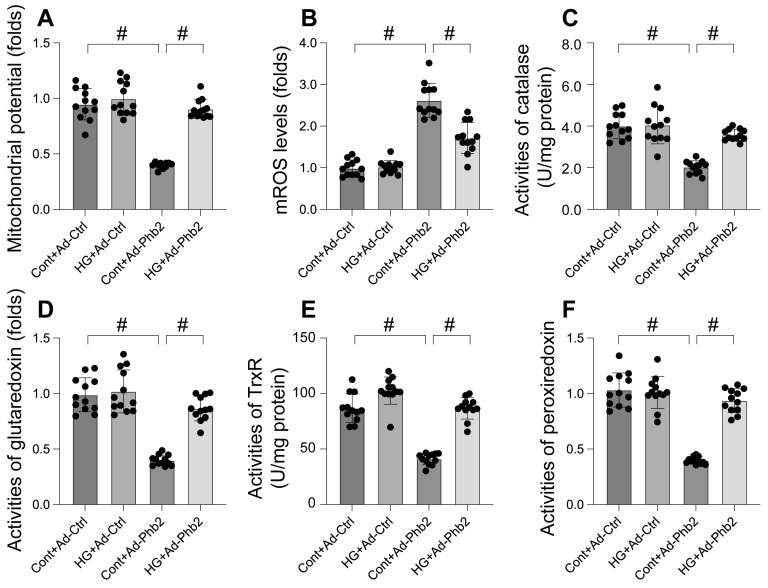
** Phb2 overexpression reduced mitochondrial dysfunction upon hyperglycemia stress. A.** JC-1 probe was used to stain mitochondrial membrane potential. B. ROS production was measured in cardiomyocytes. Phb2 adenovirus were transfected into cardiomyocytes before hyperglycemia treatment. **C-F.** The activities of catalase, glutaredoxin, thioredoxin reductase (TrxR), and peroxiredoxin were determined by ELISA. Phb2 adenovirus were transfected into cardiomyocytes before hyperglycemia treatment. #p<0.05.

This study aimed to elucidate the protective role of Prohibitin 2 (Phb2) against mitochondrial dysfunction during hyperglycemia-induced stress in cardiomyocytes. To this end, cardiomyocytes were transfected with Phb2 adenovirus to overexpress this protein. Mitochondrial function was then reassessed, particularly focusing on mitochondrial membrane potential using the JC-1 probe. Our findings, as illustrated in Figure 5A, reveal that while hyperglycemia significantly impaired mitochondrial membrane potential, this detrimental effect was mitigated by Phb2 overexpression. Additionally, we observed a marked increase in mitochondrial reactive oxygen species (mROS) levels upon hyperglycemia exposure, which was notably counteracted in cardiomyocytes transfected with Phb2 adenovirus (Figure 5B). Furthermore, hyperglycemia led to a reduction in key mitochondrial antioxidant enzymes, including catalase, glutaredoxin, thioredoxin reductase (TrxR), and peroxiredoxin (Figure 5C-F). Intriguingly, Phb2 overexpression restored these enzymes to near-normal levels (Figure 5C-F). Collectively, these results underscore the vital role of Phb2 in safeguarding mitochondrial function during hyperglycemic stress, thereby highlighting its potential as a therapeutic target in the context of cardiometabolic diseases.

### Phb2 overexpression maintained cardiomyocyte viability and function upon hyperglycemia stress

**Figure 6:**
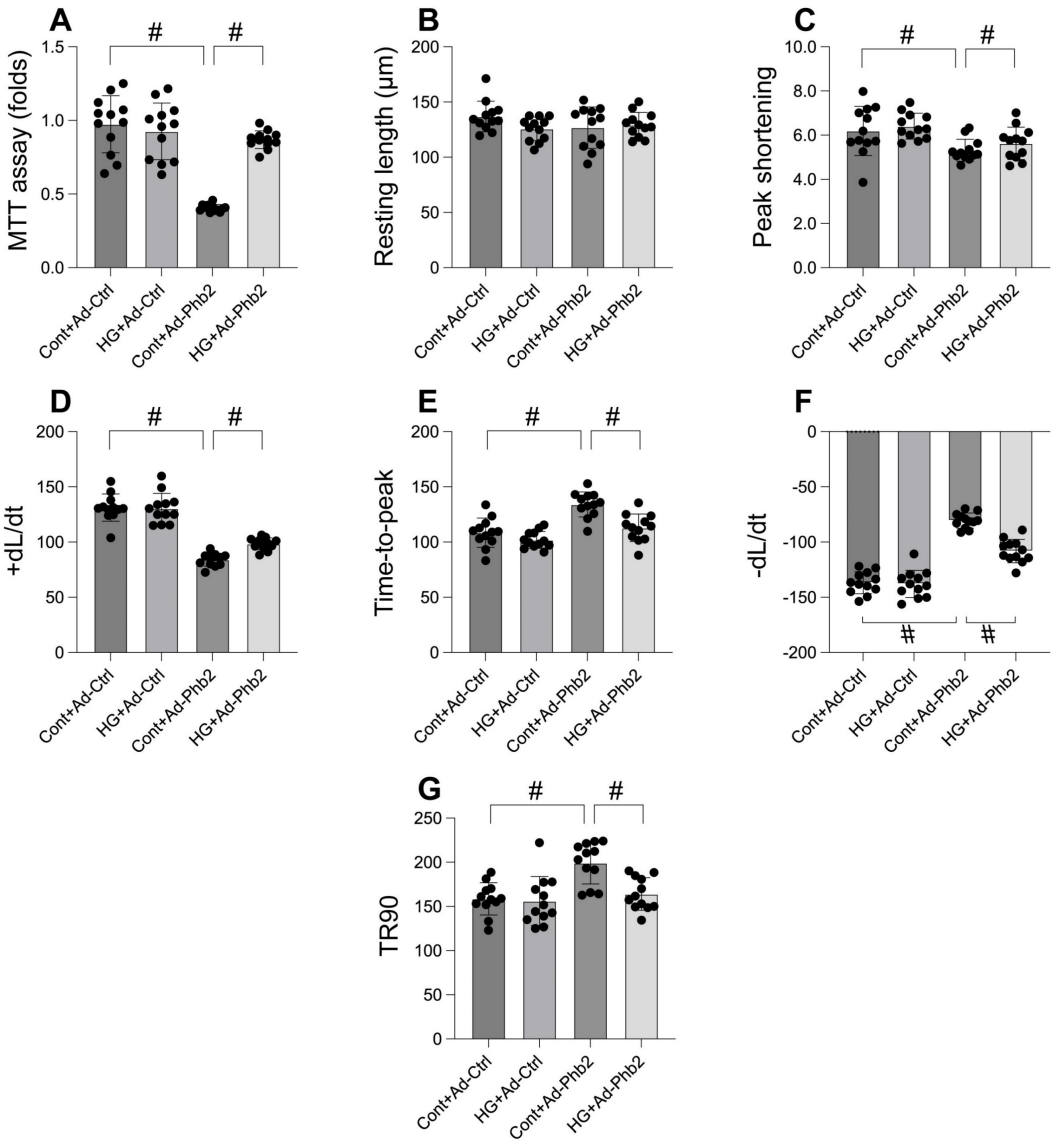
** Phb2 overexpression maintained cardiomyocyte viability and function upon hyperglycemia stress. A.** Cell viability was determined by MTT assay. Phb2 adenovirus were transfected into cardiomyocytes before hyperglycemia treatment. **B-G.** Pgam5 siRNA was transfected into cardiomyocytes and then the contractile was measured, including resting length remained, peak shortening (PK), maximal velocity of shortening (dL/dt), and time-to-peak shortening, maximal velocity of relengthening (-dL/dt), and time-to-90% relengthening (TR90). Phb2 adenovirus were transfected into cardiomyocytes before hyperglycemia treatment. #p<0.05.

Our investigation aimed to determine the efficacy of Prohibitin 2 (Phb2) overexpression in preserving cardiomyocyte viability and function under hyperglycemic stress. Utilizing the MTT assay, we observed that hyperglycemia significantly compromised cardiomyocyte viability, a deleterious effect that was substantially reversed upon Phb2 adenovirus transfection (Figure 6A). Further analysis of contractile parameters in single cardiomyocytes revealed that while resting length remained unchanged, hyperglycemia impaired contractility, as evidenced by reduced peaking shortening, maximal velocity of shortening, and time-to-peak shortening (Figure 6B-G). Additionally, abnormal relaxation kinetics were noted, including decreased maximal velocity of relengthening and prolonged time-to-90% relengthening (Figure 6B-G). Intriguingly, Phb2 overexpression ameliorated these contractile and relaxation impairments (Figure 6B-G). These results indicate that Phb2 plays a protective role in maintaining cardiomyocyte health under hyperglycemic conditions, suggesting its potential as a therapeutic target for hyperglycemia-induced cardiac dysfunction.

## Discussion

This manuscript focuses on the role of Pgam5 and Phb2 in hyperglycemia-induced myocardial dysfunction. Key findings include the observation that Pgam5 knockdown attenuates hyperglycemia-mediated cardiomyocyte death and dysfunction. The study also reveals that hyperglycemia-induced mitochondrial dysfunction is influenced by Pgam5, as Pgam5 siRNA transfection mitigates mitochondrial impairment. Additionally, Pgam5 deletion influences mitochondrial fission and mitophagy, with further investigations into how Pgam5 controls these processes through Phb2. Overexpression of Phb2 is shown to reduce mitochondrial dysfunction and maintain cardiomyocyte viability and function during hyperglycemia stress. These findings contribute to our understanding of the molecular mechanisms underpinning hyperglycemia-induced cardiac dysfunction and highlight potential therapeutic targets.

The study's focus on the roles of Pgam5 and Phb2 in hyperglycemia-induced myocardial dysfunction adds to the growing body of research linking mitochondrial dynamics to cardiac health under diabetic conditions. While previous studies have explored the individual roles of Pgam5 and Phb2 in mitochondrial function and cardiomyocyte health 15, 19, this research provides novel insights into their interplay. It elucidates how Pgam5 knockdown ameliorates hyperglycemia-induced cardiac dysfunction 27, contrasting with some studies that have not emphasized Pgam5's role in cardiac health under hyperglycemic stress. Furthermore, the study's exploration of Phb2's protective effects against mitochondrial dysfunction and its interaction with Pgam5 in a hyperglycemic environment presents new findings that differ from the typically isolated examination of these proteins. In summary, the research aligns with the broader theme of mitochondrial dysfunction in cardiac health while providing unique insights into the synergistic roles of Pgam5 and Phb2 in hyperglycemia-induced myocardial dysfunction.

It is important to note that Pgam5 has emerged as a critical regulator in these processes. Research has increasingly highlighted Pgam5's involvement in modulating mitochondrial dynamics, impacting both mitochondrial fission and mitophagy 15, 28, which are key processes in mitochondrial quality control and cellular health 29. The advancements in understanding Pgam5's function have revealed its unique role in various pathological conditions, including neurodegenerative diseases and metabolic disorders 13, 14, 30. These findings indicate that Pgam5's modulation of mitochondrial dynamics is a complex and crucial aspect of mitochondrial health, offering potential therapeutic targets in diseases associated with mitochondrial dysfunction. This synopsis brings together the recent advancements and existing knowledge in the field, shedding light on the multifaceted role of Pgam5 in mitochondrial biology.

Phb2 (Prohibitin 2) is increasingly recognized for its crucial role in mitochondrial dynamics, specifically influencing mitochondrial fission and mitophagy processes 19, 31. Research has illuminated its importance in maintaining mitochondrial integrity and function, which is vital for cellular health and homeostasis 27. Studies have delved into how Phb2's dysregulation can contribute to various pathological conditions, suggesting its potential as a therapeutic target. By synthesizing these insights, we gain a broader understanding of Phb2's multifaceted role in mitochondrial biology and its implications in health and disease.

Mitochondrial fission and mitophagy are crucial processes in mitochondrial quality control, significantly impacting cardiac health in diabetes 32, 33. Studies have shown that dysregulation in these processes contributes to the pathogenesis of diabetic cardiomyopathy, including altered energy metabolism, increased oxidative stress, and cardiomyocyte apoptosis 34-36. The role of key regulatory proteins and pathways involved in mitochondrial dynamics has been increasingly explored, providing a deeper understanding of the disease mechanism and potential therapeutic targets 37-39. This comprehensive overview combines recent advancements and existing knowledge, highlighting the critical role of mitochondrial dynamics in the context of diabetic cardiomyopathy.

When reflecting on the constraints inherent in this research, it is vital to recognise a multitude of dimensions. Firstly, while the study provides crucial insights into the roles of Pgam5 and Phb2 in diabetic cardiomyopathy, it primarily relies on in vitro models. The translation of these findings to in vivo scenarios, particularly in human physiology, remains to be established. Furthermore, the complexity of diabetic cardiomyopathy involves multifaceted pathways and factors beyond the scope of this study. Thus, while the manuscript offers significant contributions to understanding mitochondrial dynamics in the context of diabetes, further research is needed to fully comprehend and validate these mechanisms in a broader physiological context.

## Supplementary Material

Supplementary figures and tables.

## Funding

This study is supported by the National Natural Science Foundation of China (No. 82200483), the Natural Science Foundation of Guangdong Province (No. 2023A1515011687), and Guangzhou Science and Technology Plan Project (No. 2023A03J0697).
