# Supplementary material for: Pgam5 aggravates hyperglycemia-induced myocardial dysfunction through disrupting Phb2-dependent mitochondrial dynamics
Source: Int J Med Sci. 2024 May 5;21(7):1194–203. doi: 10.7150/ijms.92872 (PMC11134593; doi:10.7150/ijms.92872)
Supplement: Supplementary file 1 — Supplementary figures and tables. [file ijmsv21p1194s1.pdf]

Supplemental figures and tables for

Supplemental Figures

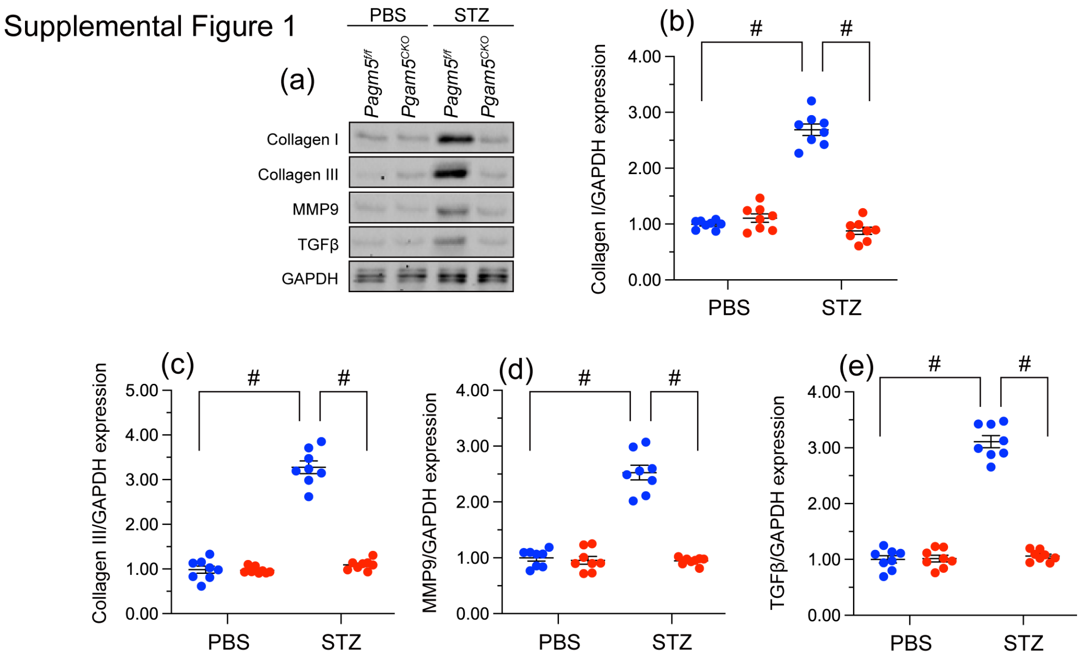

Figure S1. Cardiac PGAM5 expression is upregulated by hyperglycemia and contributes to metabolic disorder. *In vivo*, the cardiomyocyte-specific *Pgam5* knockout (*Pgam5*^{CKO}) and *Pgam5*$^{f/f}$ mice were injected intraperitoneally with STZ (50mg/Kg dissolved in 0.1mol/L citrate buffer) for five consecutive days to induce diabetes. Non-diabetic mice were the age- and sex-matched, which injected with the same volume PBS. **(a-e)** Western blot analysis of collagen I, collagen III, TGFβ, and MMP9 expression in cardiac tissue. Values are presented as mean ± SEM; For biochemical determinations, n = 6 mice per group. P<0.05.

Supplemental Figure 2

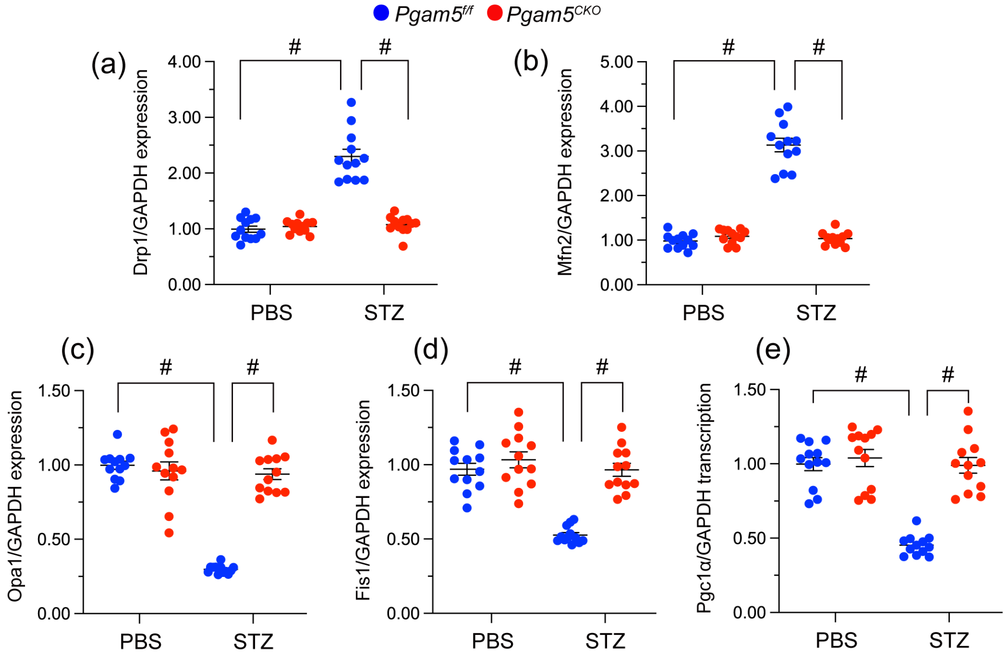

Figure S2. PGAM5 deletion normalizes MQS in cardiomyocytes exposed to hyperglycemia. *In vivo*, the cardiomyocyte-specific *Pgam5* knockout (*Pgam5*^{CKO}) and *Pgam5*$^{f/f}$ mice were injected intraperitoneally with STZ (50mg/Kg dissolved in 0.1mol/L citrate buffer) for five consecutive days to induce diabetes. Non-diabetic mice were the age- and sex-matched, which injected with the same volume PBS. **(a-e)** qPCR analysis of relative expression of *Drp1*, *Fis1*, *Mfn2*, *Opa1*, and *Pgc1α* in cardiac tissue. Values are presented as mean ± SEM. For *in vivo* data, n = 6 mice per group. P<0.05.

Supplemental Figure 3

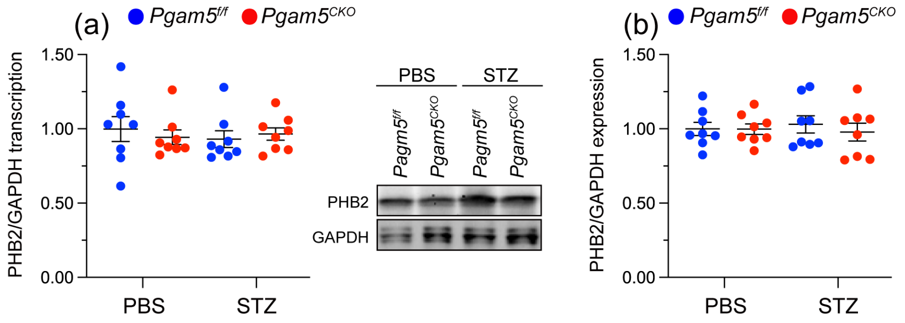

Figure S3. PGAM5 has no influence on PHB2 transcription and expression. Mouse HL-1 cardiomyocyte cell line were cultured under high-glucose medium (30 mmol/L glucose) for 48 hrs to induce hyperglycemia damage *in vitro*. HL-1 cells treated with normal-glucose medium (5.5 mmol/L glucose) was used as the control group. **(a)** Analysis of relative transcription levels of *Phb2* in cardiac tissues by qPCR. **(b)** Western blot analysis of PHB2 expression in heart tissues. Values are presented as mean ± SEM. For *in vivo* data, n = 6 mice per group. P<0.05.

Supplemental Figure 4

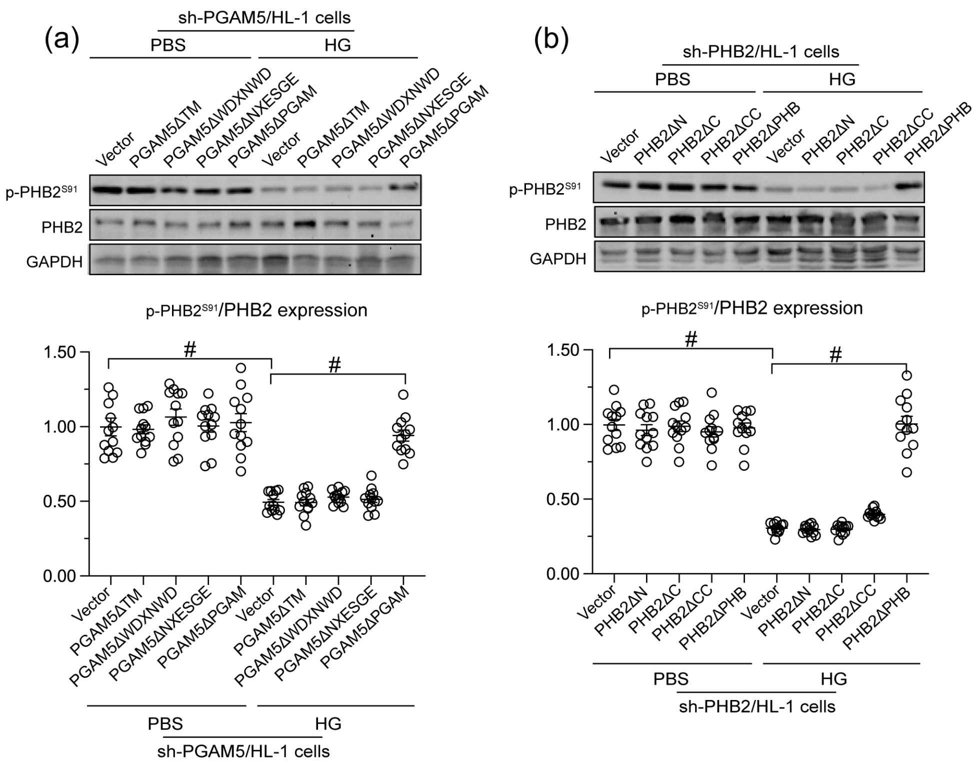

Figure S4. PGAM5 binds and dephosphorylates PHB2. Mouse HL-1 cardiomyocyte cell line were cultured under high-glucose medium (30 mmol/L glucose) for 48 hrs to induce hyperglycemia damage *in vitro*. HL-1 cells treated with normal-glucose medium (5.5 mmol/L glucose) was used as the control group. **(a)** Western blot analysis of p-PHB2^{S91} expression in HL-1 cells transfected with region-specific PGAM5 mutants. The expression of p-PHB2^{S91} was normalized to that of total PHB2. **(b)** Western blot analysis of p-PHB2^{S91} in HL-1 cells transfected with region-specific PHB2 mutants. The expression of p-PHB2^{S91} was normalized to that of total PHB2. Values are presented as mean ± SEM. For *in vivo* data, n = 6 mice per group. For *in vitro* data, n = 4 independent experiments. P<0.05.

Supplemental Figure 5

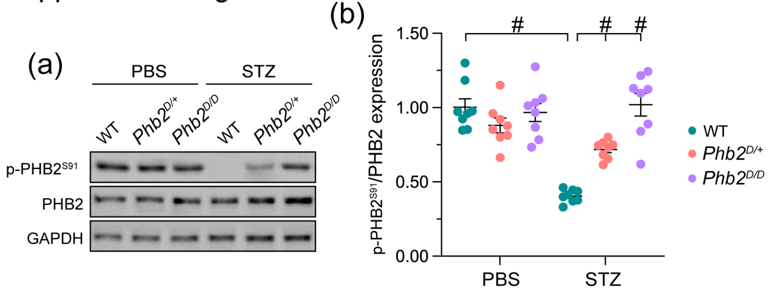

Figure S5. ***In vivo*** **expression of a PHB2^{S91} phosphorylation mutant confers resistance to DCM.** WT, heterozygous *Phb2S91$^{D/+}$*, and homozygous *Phb2S91$^{D/D}$* mice (n = 6 per type) were subjected to STZ-induced diabetes. **(a, b)** Western blots analysis of p-PHB2^{S91} expression in cardiac samples. Data were normalized to total PHB2 expression. Values are presented as mean ± SEM from 6 mice per group. P<0.05.

Table S1: Antibody information in Western blot

Name	Catalogue number	Dilution factor
Drp1	Abcam, #ab184247	1:1000
Fis1	Abcam, #ab156865	1:1000
Mfn2	Abcam, #ab124773	1:1000
Opa1	Abcam, #ab42364	1:1000
GAPDH	Abcam, #ab8245	1:1000
Tom20	Abcam, #ab186735	1:1000
Collagen I	Abcam, #260043	1:1000
Collagen III	Abcam, #7778	1:1000
TGFβ	Abcam, #215715	1:1000
MMP-9	Abcam, #228402	1:1000
PGAM5	MyBioSource, #MBS9612114	1:1000
PHB2	Cell Signaling Technology, #14085	1:1000

Table S2: Primers for qPCR

Gene	Forward Prime	Reverse Prime
Pgc1α	5′-CGGAAATCATATCCAACCAG-3′	5′-TGAGGACCGCTAGCAAGTTTG-3′
Nrf2	5′-CCTCGCTGGAAAAAGAAGTG-3′	5′-GGAGAGGATGCTGCTGAAAG-3′
Tfam	5′-GGCGAATTCCTCGAGGCCACCATG GCGCTGTTCCGGGGAATGT-3′	5′- CATACGCGTATGCTCAGAGATGTC TCCGGATCGT -3′
Pgam5	5′-GAACTACATCCACCGAGCTGA-3′	5′-GGGAAACTGCAACGCTCTAC-3′
Tgfβ	5′-TACCATGCCAACTTCTGTCTGGGA-3′	5′-ATGTTGGACAACTGCTCCACCTTG-3′
Gapdh	5′-ACGGCAAATTCAACGGCACAGTCA-3′	5′-TGGGGGCATCGGCAGAAGG-3′
Mmp9	5′-CCATCGATTAGAAGCAGGAGGACCCGA-3′	5′-GGACTAGTTGGCTAACGCTGCCTTTG-3′
Phb2	5′-AGCAGGAACAGCACAGAAGA-3′	5′-CGGAGCTTGATATAGCCAGGAT-3′
